# Leaving no one behind: how women seize control of wheat–maize technologies in Bangladesh

**DOI:** 10.1080/02255189.2019.1650332

**Published:** 2019-08-29

**Authors:** Cathy Rozel Farnworth, Tahseen Jafry, Siddiqur Rahman, Lone B. Badstue

**Affiliations:** a Pandia Consulting, Münster, Germany; b School of Engineering and Built Environment, Glasgow Caledonian University, Glasgow, UK; c Department of Anthropology, Jahangirnagar University, Dhaka, Bangladesh; d Gender and Social Inclusion, International Maize and Wheat Improvement Centre (CIMMYT), El Batán, México

**Keywords:** Sustainable development goals (SDGs), Bangladesh, Santal indigenous people, wheat–maize innovations, gender

## Abstract

Bangladesh is strongly committed to the “leave no one behind” principle of the UN’s Sustainable Development Goals. However, social norms and institutional biases in agricultural organisations can prevent indigenous peoples and women from participating in wheat–maize innovation processes, as they rarely meet the requisite criteria: sufficient land, social capital or formal education. The GENNOVATE (Enabling Gender Equality in Agricultural and Environmental Innovation) research initiative in Bangladesh shows that indigenous Santal women are obtaining access to and benefiting from wheat–maize innovations, enabling low-income Muslim women to benefit as well.

## Résumé

Le Bangladesh est fortement attaché au principe que personne ne soit « laissé pour compte », l’un des Objectifs de développement durable de l’ONU. Cependant, certaines normes sociales et biais institutionnels au sein de organisations agricoles empêchent parfois les Autochtones et les femmes de prendre part à des processus d'innovations relatives à la culture du maïs et du blé, car ils répondent rarement aux critères requis : terres suffisantes, capital social ou éducation formelle. Au Bangladesh, l’initiative de recherche GENNOVATE (Promouvoir l’égalité de genre dans l’innovation agricole et environnementale) montre que les femmes autochtones du Santal obtiennent accès aux « innovations blé-maïs » et en bénéficient, ce qui permet également aux femmes musulmanes à faible revenu d’en bénéficier.

## Introduction

Following the close of the Millennium Development Goals (MDGs) process, the governments of 193 countries agreed upon the “Agenda 2030 for Sustainable Development” with 17 Sustainable Development Goals (SDGs) and 169 targets in 2015 (UN General Assembly 2015). The SDG goals and targets are comprehensive because they integrate the economic, social and environmental dimensions of sustainable development (paragraph 5) and are founded on the respect, protection and promotion of human rights and fundamental freedoms (paragraph 19). In committing to Agenda 2030, UN member states:

we pledge that no one will be left behind. Recognizing that the dignity of the human person is fundamental, we wish to see the Goals and targets met for all nations and peoples and for all segments of society. And we will endeavour to reach the furthest behind first. (UN General Assembly 2015, 3)

The principle “leave no one behind” (LNOB) means that no goal is considered to be met unless it is met for everyone. Goal number 10 specifically commits to the social, economic and political inclusion and empowerment of all, irrespective of age, sex, disability, race, ethnicity, origin, religion and economic or other status (10.2). It recommends eliminating discriminatory laws, policies and practices and developing empowering legislation, policies and action (10.3). Goal number 5, supported by 24 gender targets across the SDGs, commits nations to “achieve gender equality and empower all women and girls” (Koehler 2016, SDG 5b). One mechanism of achieving this is through linking access to technology with women’s empowerment (Koehler 2016). More broadly, Target 2.3 recognises strengthening women’s roles in the agricultural economy as integral to doubling agricultural productivity and the incomes of small-scale food producers by 2030. Policy recommendations associated with this Target including promoting secure and equal access to productive resources, inputs, knowledge, financial services, markets and opportunities for value addition and non-farm employment (Koehler 2016).

Bangladesh has translated Agenda 2030 at the national level by formulating a Perspective Plan (2010–2021) and through bringing the SDGs into its seventh Five Year Plan 2016–2020 (Government of Bangladesh 2017a; 2017b). A “Whole of Society” approach involves all development partners – multi and bilateral agencies, NGOs and civil society, the private sector and media, in interpreting and implementing the SDGs. A SDGs Implementation and Monitoring Committee has been formed at the Prime Minister’s Office to facilitate the implementation of the SDG Action Plan. The country exhibits a generally positive commitment to gender equality at the policy level (Jafry 2013). However, the Global Gender Gap Index for 2016 (World Economic Forum 2016) suggests there is still some way to go, ranking Bangladesh 72nd out of 144 countries. It recorded improvements with respect to women’s political empowerment, but a widening of the gender gap with respect to women’s labour force participation and estimated earned income. These mixed findings are reflected in the Women in Agriculture Empowerment Index (WEAI) for Bangladesh (Sraboni, Quisumbing, and Ahmed 2013).

A literature review of how various cultural norms in Bangladesh can contribute to leaving specific groups behind (Farnworth and Jahan 2014) found a tendency in some studies to generalise, in particular relying on what people say is happening rather than examining what is actually happening. This hampers analytic clarity on the interaction between different drivers of marginalisation. The situation is made more complex by lack of agreement on the number of different ethnicities in the country, with the government recognising 27 but others claiming 45 or more (Pant et al. 2014).

In this article, we examine the drivers of marginalisation affecting the indigenous Santal people, the largest marginalised group in the country. We then examine the extent to which these drivers overlap with those affecting ethnic Bengali women from poor and middle-income groups and assess the implications of relatively greater personal freedoms – for example, strong mobility experienced by Santal women – for their ability to reach out to ethnic Bengali women and help them collectively seize control over development processes.

To achieve our objectives, we examine the frameworks that suggest analytic pathways for conceptualising marginalisation processes. Mittal, Pereram, and Korkeala (2016) outline four structural drivers underpinning marginalisation processes: (1) an inadequate asset base – natural, physical, financial, human, social and cultural; (2) poor access to services and infrastructure – health, energy, water, transport and markets; (3) weak political voice, empowerment and institutional governance; and (4) identity-based discrimination. The converse of the first three structural drivers – assets, services and voice – equally act as “enablers” to lift people out of poverty. This is not true, the authors argue, of the fourth driver. The processes of identity-based discrimination – and the social norms which underpin and “rationalise” this – are an underlying driver hampering marginalised people from utilising the first three drivers to their advantage. This interlocking process is depicted in Figure A1 (Online Appendix).

Identity-based discrimination operates at group levels and thus contributes towards the development of group-based “horizontal” inequalities (Kabeer 2016; see also Ribot 2009). The most enduring horizontal inequalities, Kabeer argues, are those associated with identities ascribed at birth such as race, gender, caste and ethnicity. She highlights how “personal” the articulation of social hierarchies can be. Cultural norms and practices can “disparage, stereotype, exclude, ridicule and demean certain social groups, denying them full personhood and equal rights to participate in the economic, social and political life of their society” (Kabeer 2016, 13). Stuart (2016) comments that the use of the term “group-based” is valuable yet paradoxical, as its real value is in allowing us to see the individual, rather than working with a concept of the poor, as aggregate poverty numbers. Understanding how different types of group-based marginalisation overlap, layering disadvantage upon disadvantage – for example, being poor, aged, a woman, a widow and a member of a discriminated-against ethnic minority – enables us to perceive almost viscerally what it means to be left behind and how difficult it can be to escape that situation (Mittal, Pereram, and Korkeala 2016).

Conventional approaches to poverty reduction, which may focus on alleviating a specific type of inequality such as age or disability-based discrimination, have often been ineffective in transforming the situation of LNOB people because each of the overlapping inequalities they experience fuses with, and can exacerbate, the effects of the other inequalities, making it particularly difficult to develop pathways out of poverty (Kabeer 2016). In particular, an apparent lack of “productive assets” may mean that marginalised people are not targeted in mainstream development projects. Rural advisory services and agricultural research organisations often select farmers with a specified minimum land area and other assets to trial innovations, for example improved seed, machinery and other agricultural practices (Bellon, Hodson, and Bergvinson 2005). This can, by default, exclude marginalised communities. Since they are not included in training, their potential to benefit from innovations is diminished, thus leaving them further and further behind. Their marginalisation is systemic and systematic.

However, this process can be counteracted. This article provides insights into how layers of overlapping disadvantage are being challenged in one community in northern Bangladesh. The hypothesis guiding our empirical research and analysis is that although the wheat–maize innovation processes in the community are primarily directed at middle-income male farmers, women from different income classes and ethnicities are seeking inclusion. The research questions are: (1) what strategies do women develop to participate in innovation processes as individuals and through organisations? and (2) what do women gain from securing inclusion? Collectively, these research questions set out to explore how women deploy their agency to lift themselves out of poverty by securing a role in wheat–maize innovation processes and whether these strategies differ depending on the type of marginalisation – as women, indigenous minorities or low and middle-income farmers. In the conclusion, we build on Kabeer’s (1999) work to define women’s empowerment through the concepts of agency and power to help us interpret the findings. We expect this analysis and findings to help understand how agricultural research partners can work to strengthen the contribution and voices of the women who have long experienced differing forms of marginalisation; and support their resistance.

## Marginalisation and resistance processes affecting Santals in Bangladesh

In our discussion, we examine the ways in which the norms and practices emanating from the dominant socio-political classes in Bangladesh, which “disparage, stereotype, exclude, ridicule and demean certain social groups” (Kabeer 2016; see also Kelkar 2009), are extended towards indigenous peoples, particularly Santals, in the country. Evidence of Santal resistance to marginalisation processes is presented, followed by socio-economic data which nevertheless demonstrates the serious consequences of marginalisation for Santali development indicators. Sex-disaggregated data compares the situation of low and middle-income ethnic Bengali women and men to that of Santali in the same locations. This overview provides contextual information within which our research data can be assessed and understood. The special focus on Santals, rather than an equal focus on ethnic Bengali and Santals, is necessary to highlight additional features that serve to marginalise Santals. These do not apply to ethnic Bengalis.

The politics of the Bangladesh post-colonial state, which sees itself as Muslim and Bengali, has served to render indigenous people “ubiquitously absent as subjects, as actors, as agents in the national imagination; they are invisible in the administrative, educational, economical systems, and in the popular media” (Priyadarshini 2012, 22; see also Karim 1998). The term “indigenous” is not officially recognised; with the government declaring that other ethnicities (in Bengali *upojati*, which means small ethnic group) only entered the country in the sixteenth century (Alam 2015). Substituting the terminology of indigenous peoples with small ethnic groups implies that the state does not need to follow international conventions with regard to acknowledging and respecting the rights of indigenous peoples. Indeed, the celebration by Santals and others of the International Day of the World’s Indigenous Peoples was not permitted for some years after 2011 (Alam 2015). Celebration is now permitted in Dhaka, though not as a national event. The Bangladesh Adivasi Forum, the Bangladesh Indigenous Peoples Forum and other civil society groups seek recognition in the Constitution and are members of Citizens’ Platform for the SDGs (http://bdplatform4sdgs.net).

Indigenous women, alongside ethnic Bengali women, have a long history of active militancy and passive resistance in Bangladesh. Alam (2015) recounts how Santal and low-income ethnic Bengali women defied armed government troops during a demonstration against proposed mining activities in Phubari on 26 August 2006. Male demonstrators fled but women chased armed troops with choppers and brooms and blocked entrances to the village. The next day women organised a large demonstration at Phulbari municipality. The image of Santal and ethnic Bengali women working together to fight for the survival of their community drew the attention of the nation to the struggle. A national leader explained, “usually [women] do not participate in regular meetings but whenever there is an emergency they never hesitate because they have stronger feelings than men for livelihood, house, children and other kin relations” (Alam 2015, 42–43). Women also resisted by refusing to provide information on households to mining company members and chasing them away (Alam 2015). Santal women were part of earlier struggles such as the Tebhaga movement of indigenous sharecroppers, particularly in northern Bangladesh (our study location) to reduce the share given to powerful landowners from one-half to one-third (Hashmi 1994, as cited in Alam 2015). Indigenous and ethnic Bengali women were active militants and fought alongside men in the Bangladesh Liberation War of 1971 (Harrington 2013). Harrington argues that male-dominated narratives of this war still find it too difficult to assimilate the transgressive nature of women guerrilla fighters, preferring to cast them as war heroines (*birangona*), defined as women who were sexually victimised as a consequence of the struggle, rather than as active fighters.

Despite resistance struggles, the negative consequences of systemic discrimination over decades are evident. Brandt (2011) notes that Santal indigenous people have complex religious beliefs and are subjected to considerable missionary efforts by the Seventh-day Adventist Church. Whilst these efforts are placing a strain on Santal indigenous religion, she observes that missionaries are often the only institution interacting with the Santal (Brandt 2011; see also Gauri and Galel 2005; and Samad 2006). Priyadarshini (2012) contends that indigenous women in Bangladesh are sexually, economically and politically discriminated against by some ethnic Bengali. At the same time, indigenous women face gender-based discrimination and violence within their own households and communities. They have few inheritance or parental rights and do not participate equally in religious rituals (Priyadarshini 2012).

Santals score very low on multiple indicators including health, educational level and land ownership (Abdullah 2014; Uddin 2009; Hossain and Tollefson 2007). Samad (2006, 9) argues that the effects of discrimination – including land-grabbing, threats, evictions and killing – are such that Santals are at a considerable disadvantage compared to some other indigenous groups as well as the majority ethnic Bengali population. Access to education and Santal language publications can be difficult and many Santali children do not attend school or perform poorly, due to their poor knowledge of Bengali (Cavallaro and Rahman 2009). A study conducted in six villages showed that over a period of 50 years Santal families holding more than 15 acres of land diminished from 72 to zero, partly due to poor understanding by Santals of their rights under land law and “shrewd exploitation” (Rahman and Bhuiyan 2008). The study comments dismally that the Santal are “losing their existence with regards to their economic, social and political lives” and that they now work as day labourers on the land they previously owned (Rahman and Bhuiyan 2008, 4). A comparative study of 288 couples (145 Muslim and 143 Santal) in one village (Uddin 2009) found significant differences in educational attainment, occupational status and income between Muslims and Santals. The study further recorded gender differences with respect to these indicators between women and men within each ethnicity, and between each ethnicity, as shown in Table 1.

**Table 1.** Selected indicators, Santal and Bengali couples in same village.

	Santal couples	Santal men	Santal women	Bengali Muslim couples	Bengali Muslim men	Bengali Muslim women
Low-income	80%	–	–	33%	–	–
Middle-income	16%			27%	–	–
High-income	4%	–	–	40%	–	–
Never been to school	–	68%	72%	–	30%	41%
Farmer on own land	–	5.5%	–	–	62%	–
Day labourer	–	84%	90%	–	12%	3.5%
Housewife	–	–	5.6%	–	–	92%

Source: Uddin (2009).

Note: Percentages rounded up and down.

Table 1 shows that income differentials are sharp, with considerably more Santal couples in the low-income bracket, and very few in the middle to high-income brackets. Bengali couples are distributed more evenly across the three income bands. There is a strong differential in the number of men farming their own land. Low land ownership and low levels of education among Santal contributes to high levels of Santal men seeking work as day labourers. Discrepancies between the genders also emerge. Among Santal, few women and men have had any formal education. However, although Bengali Muslims are more educated, the gender gap between women and men on this indicator is more marked. The most startling difference between Santal and Bengali women is in occupation, with nine out of 10 Santal women in daily wage labour and nine out of 10 Bengali women remaining at home.

Figure A2 highlights these interrelationships diagrammatically. It shows how constraints that typically apply to ethnic Bengali women also apply to Santal women, such as time-consuming responsibility for household and care work, and an inability to sell agricultural produce at formal markets. These constraints are shown in blue. However, the position of Santal women is worsened through identity-based discrimination, the loss of land, general lack of targeting in development programmes and even lower literacy than the average for Muslim women. Constraints specific to Santal women are shown in orange. Low-income Muslim women share some constraints with Santal women, particularly engagement in low-paying day labour. The constraints shared by Santal and low-income Muslim women are shown in green. These drivers of marginalisation can act together to create a downward spiral, whereby negative outcomes cause further negative outcomes. The outcomes are unevenly distributed among women, with Santal women experiencing the most intense overlapping of drivers.

## Method and materials

### Methodology

The data used in this article is derived from GENNOVATE (Enabling Gender Equality in Agricultural and Environmental Innovation). This is a cross-CGIAR (Consultative Group for International Agricultural Research) initiative examining how interactions between gender norms, agency and other contextual factors shape access to, adoption of and benefits from agricultural innovations in rural communities worldwide (see more details on the Gennovate methodology in Petesch, Badstue, and Prain [2018]). Data collection in Bangladesh took place in 2015.

GENNOVATE uses a comparative case study approach deploying standardised instruments to identify factors that hinder or facilitate and promote men and women’s individual and collective capacities for engaging in innovation processes. The methods are qualitative sex-segregated focus group discussions (FGD) and semi-structured interviews (SSIs) with participants of different ages. The adult FGDs are sub-divided by economic class with respondents drawn from poor and middle-income categories using locally developed classifications. One-on-one interviews are held with locally recognised innovators. Landless women and men, who neither own nor lease land, are not included as a analytic category although they may be active in agriculture-related occupations. In all cases the facilitators and note takers are of the same gender as the respondents. The tools are summarised in Table A1 (Online Appendix).

The FGD questionnaires comprise structured checklists to enable international comparisons between responses to be made with some scope for probing. Thus the questionnaires are not tailored to specifically enquire into the situation of indigenous peoples. However, since they allow some additional probing within the strict format, Bangladesh enumerator teams were able to explore differences between cultural norms, religious beliefs and other factors relating to innovations of FGD members. In this case study, participants in each FGD comprised of people from the Santal community, Bengali Muslims and Hindus. A total of 27 people from the Santal community were interviewed (75 respondents in total). Table 2 provides a breakdown of the distribution of respondents by research method and by gender and ethnicity/religious affiliation.

**Table 2.** Breakdown of respondents by research method and by gender and ethnicity/religious affiliation.

Research method^a^	Santal women	Santal men	Muslim women	Muslim men	Hindu women	Hindu men
Two low-income FGDs	4	5	6	6	0	0
Two middle-income FGDs	4	3	5	8	1	1
Two youth FGDs	6		4	8	–	1
Eight innovator pathway/life history SSIs	1	2	3	2	–	–
Two community profile SSIs	1	2	1	3	–	–
Totals (individuals)	16	11	19	27	1	1

^a^FGD: focus group discussion; SSI: semi-structured interview.

Note: See Table A1, Online Appendix, for details on research method.

The data was collected by a team from Global Communication Centre, Grameen Communications, BANGLADESH, and was translated into English by an externally hired translator. NVivo Qualitative Software was used to conduct the initial variable-oriented analysis. This permitted the identification of emergent themes within and across the data. This was followed by systematic in-depth data analysis.

### Research site, target groups and key innovation institutions

Kalipara (a pseudonym) is a village located on Bangladesh’s northern plains. Agriculture is central to its economy. Rice, maize, wheat, dal, jute, mangoes, lychees and vegetables are important crops. Livestock, including goats, cattle, poultry and fish are widely raised. Over the past 10 years, maize and especially wheat have become popular commercial crops. New technologies, including improved seeds and breeds, machinery and new methods of cultivation have been introduced in almost all crops and livestock. The development of paved roads enables male farmers to sell produce in local towns, as does the widespread use of cell phones. Electrification and the internet have also helped to improve livelihoods. As a consequence, Kalipara has been experiencing important economic dynamism for around a decade. Many men now work in off-farm occupations, and some farmers are becoming wealthy as a consequence of all these changes. Since there have been so many changes, it is not possible to directly attribute improvements in livelihoods specifically to the adoption of wheat–maize innovations. At the same time, successful adoption of these technologies demonstrably plays a role in economic betterment as shown in the Findings.

It was not possible to obtain nuanced data on relative income levels by ethnic group or religious affiliation. However, the Ladder of Life activity conducted as part of the Well-being FGD suggests that 40 per cent of all households in Kalipari are considered very low-income. A further 25 per cent are low-income. Middle-income households comprise around 20 per cent of all households, and 15 per cent of all households are categorised as wealthy.

In Kalipara, the situation of the Santals initially appears much as presented in Table 1 above and Figure A1 in the Online Appendix. Santals once dominated the village numerically. Today they comprise approximately 20 per cent of the population due to immigration by Bengalis and most Santals have become Christian. Bengali Muslims account for 75 per cent and Hindus – 5 per cent^1^of the population. Key informants claimed that the overwhelming nature of Bengali immigration, combined with low literacy rates among Santals, resulted in the latter losing almost all their land to Bengalis. Today, Santals hold less than 10 per cent of the land. The majority of Santal women and men work as day labourers, as do many low-income Muslim women and men.

Respondents explained that, under Islamic law, daughters, as well as sons, can inherit land, though in reality daughters rarely claim their inheritance. Santal norms generally hamper women from inheriting land and they face other normative restrictions as noted by Priyadarshini (2012) above. Santal and Muslim women are responsible for house and care work, though, unlike Muslim men, Santal men help their wives from time to time in all tasks except childcare. Women from both communities also farm vegetables around the house, raise cattle and goats, and conduct post-harvest processing of field crops like rice and maize.

Santal women experience considerable, though not total, mobility. This is in sharp contrast to Muslim women, particularly from the middle-income groups, who experience very little mobility and are not expected to work in the fields. Santal women work alongside men, and by themselves, in the fields on crops such as rice, wheat and maize. A middle-income Muslim woman commented, “Married Santal women have freedom to participate both in field and home. These women are participating at all levels of agricultural activities.” Cultural norms prohibit Bengali and Santal women from selling agricultural produce in local markets, though they sell vegetables, goats and cattle from the farm gate to middlemen. Although women can obtain credit from NGOs, this money is often turned over to men for their use.

### Partners for wheat–maize innovation processes in Kalipara

The International Wheat and Maize Improvement Centre, CIMMYT, has a strong presence in Bangladesh. In our case study, CIMMYT has partnered with a large NGO, Rangpur Dinajpur Rural Service (RDRS). RDRS is active in hundreds of communities across northern Bangladesh and forms farmer clubs through which it can introduce new agricultural technologies. CIMMYT piloted wheat–maize innovations through the Kalipara branch of RDRS in 2013 and rolled out the project in 2014/2015 to more farmers. The innovations included improved wheat and maize varieties, inorganic fertiliser and machinery including the power tiller operated seeder (PTOS). The PTOS is attached to a small two-wheeled tractor to simultaneously drill, sow and fertilise crops in lines.

When the RDRS started its partnership with CIMMYT, it selected individuals who had proven successful experience in earlier agricultural innovation processes. RDRS targeted farmers with whom they had strong existing relationships and who were considered enthusiastic and willing to try out wheat–maize innovations. The amount of land deemed necessary for wheat–maize innovation varies across communities, but in Kalipara RDRS stipulated that participating farmers should farm at least 20 decimals (.08 hectare), whether owned or hired in. The land had to be upland, situated next to a road in order to maximise visibility of the trials and because when machinery is driven to more remote locations, fuel costs more – thus making them unattractive to farmers at the early adoption stage. Potential participants also needed to have a minimum level of education. These criteria automatically excluded most low-income women and men, the majority of whom have never been targeted by RDRS or the government agricultural services. Even so, a few low-income Muslim men known to RDRS were targeted.

The RDRS, with funding from the European Union and other NGOs, established a Union Federation – an outreach platform specifically to reach women farmers – in Kalipara in 2013. This is just one of many Union Federations established by RDRS across its intervention area. RDRS works through the Union Federation to direct agricultural innovations specifically to women. The Kalipara Union Federation provides courses in income-generation activities like vegetable growing, sewing and hobbies such as the harmonica, and it holds regular meetings where women members can share their experiences of new technologies, including wheat–maize innovations. At the time of study the Kalipara Union Federation had a membership of 826 individuals, of whom 766 were women and 60 were men. All men members were Santals (7% of the membership). Santal women comprised 55 per cent (422 individuals) of the membership; and Bengali Muslim/Hindu – 38 per cent (344 individuals). Members were derived from Kalipara village, as well as surrounding villages. Since the Kalipara Union Federation building is physically located close to the homes of Santal people, this has promoted Santal participation as members and in the leadership. Currently, the president of the Kalipara Union Federation is a Santal woman, Joytee (a pseudonym), who was elected by Muslim and Santal women for her strong leadership skills, proven innovation expertise and personal motivation.

Male trainers from RDRS come to the Union Federation to train women in wheat–maize innovations (and other technologies). The personal link between RDRS and the Union Federation is the president of the Union Federation. When women farmers have queries about wheat–maize innovations, they approach Joytee who provides expert opinion and solicits assistance from the RDRS, if required. Women are also free to approach RDRS directly, but in most cases they will do so with the knowledge and support of Joytee.

Table A2 (Online Appendix) shows the actual percentages of farmers trained in wheat–maize innovations directly through the RDRS and through the Kalipara Union Federation. It shows that more Muslim/Hindu women (14.7% of all trainees) were trained than Santal women (12% of all trainees), although Santal women are more numerous in the membership (55%) than Muslim/Hindu women (38%). It further shows that 270 Muslim/Hindu men were trained directly by RDRS (72% of all trainees) and that only five Santal men (1.3% of all trainees) were trained (through the Union Federation). It appears that joining the Union Federation is the only way for Santal men to put themselves forward for potential targeting for wheat–maize training events. They are not targeted directly by RDRS unlike middle to high-income Hindu/Muslim men. This is presumably because all Santal men fall into the marginalised/low-income category. It would further appear that no low-income Muslim/Hindu men are targeted.

Table 3 provides an overview of beneficiaries by income band. The 105 individuals trained through the Kalipara Union Federation are low-income Santals and Muslims (100 women and 5 men), and all the middle and high-income categories of trainees (total 270) are male Muslims or Hindus.

**Table 3.** Beneficiaries of training in wheat–maize innovations, by income category.

Income category	Number of individuals (% of total)
Marginalised/low-income	105 (28%)
Middle-income	180 (48%)
High-income	90 (24%)

## Findings

The findings are presented as follows. First, we discuss differences in women’s gender interests in maize-wheat technologies by ethnicity/religious affiliation and socio-economic class. Second, we explore the drivers which facilitate or deny women opportunities to meet their gender interests. Third, we examine the strategies women use to secure inclusion in wheat–maize innovations. We do not discuss the position of low-income Santal and Muslim/Hindu men, who are not targeted for any training in wheat–maize innovations. Further research on the implications for intra-household discussion processes and for the sustainable adoption of new technologies, when low-income women but not low-income men are targeted, is required.

### Women’s gender interests in wheat–maize innovations

The gender interests of women in wheat–maize innovations vary by socio-economic position and cultural norms. Middle-income Muslim women do not work on field crops. However, they want to be able to discuss agricultural innovations knowledgeably with their husbands and to make decisions together. They have a vested interest because innovation costs money and thus has the potential to impact negatively – or positively –the family budget. Whilst in such families men are expected to earn, effective household budgeting is considered a core management responsibility for women. Women argued that if they were able to participate in training on wheat and maize and thus understand the costs and potential financial benefits of the innovations involved, they could help to “improve their household’s position”.

Low-income Muslim women have the same budgetary interests as middle-income Muslim women. However, low-income Muslim women experience substantially more freedom of movement. They work as daily hired labourers and on their family fields alongside men, and thus have clear interests in improving household income through using new technologies themselves. The use of machinery promises considerable labour savings in their own fields as well.

Santal women, both middle- and low-income, similarly experience freedom of movement, except for being able to sell in local markets, which is a taboo for all women. Santal women in households with land work in field-based agriculture and, indeed, appear to be taking over from men in many activities, as men start to seek off-farm income generation opportunities. A “good” Santal wife, according to the Wellbeing FGDs, is expected to be knowledgeable about improved seeds, inorganic fertilisers, irrigation methods and the use of herbicides and pesticides. They thus have strong vested interests in all agricultural innovation processes including wheat–maize.

### Drivers of inclusion and exclusion

Despite their interest in agricultural innovations, cultural norms frequently inhibit Muslim women, particularly middle-income, from participating in agricultural training courses in relation to wheat and maize. Such courses are in themselves not exclusionary of middle-income Muslim women, but women who do not adhere to cultural norms are at risk of social exclusion.

Middle-income Muslim women noted, sometimes bitterly, that “many men are not open to accept training for women, particularly regarding field crops” since “illiterate husbands think what will be the benefit if my wife attends agricultural trainings since she is not allowed to work in the field” (interviews with participants). They agreed that, due to norms favouring seclusion, “there is little scope to try out the new practices, but we want more opportunities which will help improve overall economic returns to the family”. They are further constrained by the fact that most agricultural extension officers are usually inaccessible to them, as women, because of the norms prohibiting interactions with non-family men: “Most agricultural extension agents are men and this is a barrier for women to attend modern training. Our husbands and families don’t let us” (interview with a participant). These findings incidentally suggest that men rarely share information from technical training with their wives, though more research is needed to verify this.

Low-income Muslim women are doubly excluded through their gender and through their low-income status. Although the RDRS and its outreach platform, the Kalipara Union Federation, are theoretically open to low-income households, in reality, the majority are unable to meet the minimum targeting criteria for training in wheat–maize innovations. Female and male low-income respondents expressed frustration and disappointment, reiterating that they want to try the innovations but do not receive training and support and do not have sufficient money to invest on their own account. They acknowledged that some poor farmers are risk-averse but argued that “many poor farmers are motivated and inspired by the new practices; the absence of support is a barrier” (interview with a participant). However, findings show that some low-income women and men have succeeded in raising themselves and their households out of deep poverty over a period of several years through determination, hard work, training in innovations like vegetable gardening and the respect and support of in-laws. This has enabled a few men to be selected for RDRS training courses on wheat–maize innovations.

A cross-cutting complicating dynamic in this community is the identification of wheat–maize innovations with Santals in the RDRS and the Kalipara Union Federation. Some early male Muslim adopters using the PTOS were criticised by other Muslim men:

We have worked so hard to plough and sow seeds on our farm but you haven’t even cultivated your land! It will never produce a crop. What kind of method have you learnt from the aboriginal people? You will never get crops from here. (Interview with a participant)

Here, the word “aboriginal” rather than Santal is intended to be derogatory. Some Muslim innovators were also accused of wanting to become Santal or Christian. Such attitudes initially hampered wider adoption among eligible Bengali Muslims with only eight men willing to try the new technologies to start with.

The technologies themselves, as suggested in the quote above, also proved a source of contention. A Muslim woman explained:

Other people discouraged us a lot. They said a lot of negative things and said things like our land won’t grow any crops because we didn’t plough it and we should just break the land and cultivate it again but we didn’t listen to them. We were quiet and carried on with our decision of continuing to practice this method. (Interview with a participant)

The data shows, in fact, that in almost every case neighbours and other community members were suspicious and negative towards the innovator regardless of their gender and ethnic/religious affiliation.

### Women’s strategies to secure inclusion in wheat–maize innovation processes

Women agreed that it is a normative must for women regardless of ethnicity to seek permission from male spouses and elders in the lineage. Santal women generally demonstrated stronger voice in decisionmaking than ethnic Bengali women, though of course there is variation among both groups. Santal women emphasised that they lead discussions with spouses and their aim is to secure agreement with their objectives. They first discuss new technologies with agricultural experts from the RDRS and the Kalipara Union Federation. They then talk to their husband. For instance, selection and purchase of improved wheat seed “is decided by a woman after discussing the matter with her husband”. A woman also “discusses with her husband to decide about how much fertiliser is to be used where on the farm”.

Securing male support entails much more than demonstrating observance of a cultural norm. All women innovators reported that wholehearted family support was critical to their willingness to take risks and try something new and they provided considerable detail of such encouragement. Some men innovators – Muslim and Santal – also stressed they talk to their wives and family members before trying something new: “the money is going to be spent from family income so discussing with wives is important”.

Some Santal women are not only taking up innovations, they are also adapting them and in this way secure respect from other farmers, men and women, Muslim and Santal. Joytee explained that when she used the PTOS, referred to as the Bednala process in Bengali, she realised that despite the labour saving advantages some technical issues remained. Some of the seeds were vulnerable to disturbance by chickens foraging for food. She also noticed that seeds germinated at different rates due to differences in soil water content and that they grew at various speeds due to differences in soil fertility. She therefore fenced her land, selectively improved soil fertility and developed an effective irrigation system. When she shared her experience with the RDRS, “they told me that we taught you our method now you can do whatever you want”. Joytee then took her experience to the Kalipara Union Federation meeting where interest was higher. “Women came to me to find out more. I told them to apply this method to get a better result. They listened to me very attentively.” More broadly, Bengali and Santal women appreciate the Bednala process (PTOS) because it saves them a lot of time (amounting to several days). They can attend to their other work and this is their “favourite thing” about the new practice.

Joytee was supported by her husband, brother-in-law and two daughters to innovate in wheat and maize. They encouraged her to try out new varieties and to participate in agricultural training courses. This included learning to drive the PTOS and operate a thresher, and now she acts as a hired machinery operator. Joytee started to access information on seeds, pesticides and market prices through her mobile phone and she went to the local market to obtain information directly from various NGOs. Whereas in 2013 Joytee and her family were among the most impoverished in the community, on the lowest rung of four according to the Ladder of Life exercise, in 2015 the household assessed itself to be on level 3 which is considered middle-income. She is sending her daughters to good colleges, has purchased quality furniture and started a savings account, and highlights that she has time for her family, as well as leisure. Joytee attributes her success to working closely with the RDRS and the Kalipara Union Federation and argues that anyone who wants to succeed must have institutional support. They cannot succeed on their own. They also have to have faith in the new technologies. Joytee’s standing in the community has risen, such that Santal, as well as ethnic Bengali Muslims, both women and men, decided to imitate her by trialling the innovations, including leasing in land in order to try them out.

A low-income Muslim woman called Bilkis (a pseudonym) is one of them. Over a period of around 10 years Bilkis accumulated funds working hard on family land and as a day labourer. She gained the respect of her in-laws for making thoughtful decisions and they now allow her to make all important decisions in her personal life and in agriculture. When she learned the Kalipara Union Federation was offering technical training on maize and wheat:

I met with the president, Aunt Joytee. After this meeting, I joined the Kalipara Union Federation and formed a group with 25 other women in our village [to work on the innovations]. Aunt Joytee helped and advised me regarding anything I wanted to discuss.

Bilkis followed the recommendations provided by RDRS through the Kalipara Union Federation and now her “yields are booming”. Several low-income Muslim women are taking out credit to lease land to trial the innovations. One explained, “the main good point of the new methods is that earlier I got 960 kg maize from 1 big ha of land [0.13 hectare: 0.33 acre] but now I am getting 1,600 kg maize from 1 big ha”; and another said, “My brother inspired me a lot. He told me that machines will bring great results but hard work is mandatory as well.”

## Discussion

As mentioned earlier, the hypothesis guiding the research and analysis of the GENNOVATE data is that although wheat–maize innovations are directed at middle-income male farmers, women from different income classes and ethnicities are seeking inclusion. The research set out to determine: (1) the strategies women develop to participate in innovation processes as individuals and through organisations; and (2) the benefits women gain from securing inclusion. We now ascertain the degree to which the hypothesis and research questions have been answered. In the Conclusion we set our findings in the overall context of the SDGs and the ambition to “leave no one behind”.

The research hypothesis has been substantiated. The data show that women have strong vested interests in wheat–maize innovation processes, though the precise form of that interest differs according to locally specific cultural norms which shape their lives as low- and middle-income female farmers and whether they are Santal or Muslim. As a consequence of these differences, the strategies women have developed to access and participate in male-dominated innovation processes differ.

**Figure 1.**
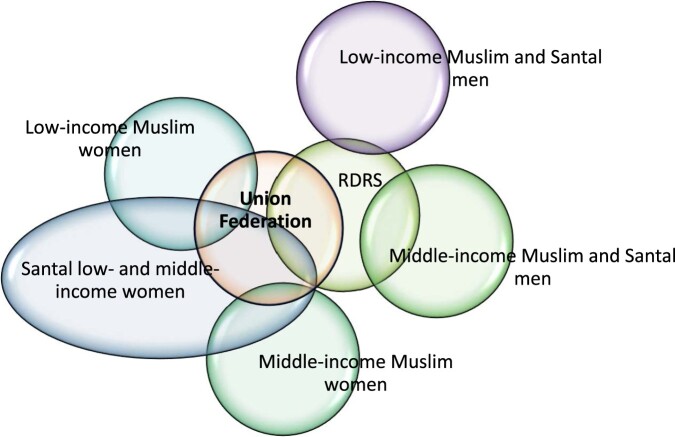
Key actors and institutional relationships, partly shaped by strategies women develop to access and participate in wheat–maize innovation processes. Source: The authors. Note: RDRS: Rangpur Dinajpur Rural Service.

Figure 1 is a Venn diagram which visually pulls together the key findings in relation to the first research question – the strategies women develop to participate in innovation processes as individuals and through organisations. It depicts the key actors and their interrelationships with each other. The points at which boundaries overlap show where information-sharing on wheat–maize innovations is occurring. This is shown to be a strongly gendered process. The woman-dominated Kalipara Union Federation lies at the centre of the diagram. Its presence is decisive to the ability of women to develop and realise their strategies. The Kalipara Union Federation is an effective mechanism for RDRS with its male extension officers to train women in women-only groups in wheat–maize innovations. Santal women have been the primary beneficiaries. They have profited because they are strongly involved in all aspects of field crop agriculture, are physically mobile and because they have demanded inclusion. The success of Santal women has in turn inspired some low-income Muslim women to become members of the Kalipara Union Federation and to be trained in wheat–maize innovations themselves. That is to say, women who have been ethnically marginalised and those who have been marginalised due to their poverty have interests in common. Joytee, the Santal woman president, is an inspirational role model for such women. The least successful would-be participants in innovation processes are middle-income Muslim women. Their desire for training in wheat–maize innovations has yet to be met due to lack of support from male family members – though a few of such women receive training on vegetable production and other forms of income generation at the Kalipara Union Federation. This suggests that cultural norms rather than ethnic identity shape their opportunities. Figure 1 further shows that RDRS interacts directly with middle-income Muslim and Santal men, and only very marginally with low-income Santal and Muslim men. Potential information-sharing relationships between low- and middle-income women and men were not explored in this study and are thus not depicted.

The second research question enquires into the benefits that women gain from inclusion. The data is less rich on this but it suggests benefits can be large. Successful women innovators reap respect from their spouses and extended families, and from the broader community. Within the household this can translate into stronger participation into intra-household decisionmaking processes. Improved income means women start to achieve important household and personal goals. Women also save time because the new technologies are labour-saving.

## Conclusion

Our research shows that Muslim and Santal women in the study community continue to face institutional barriers to accessing agricultural technologies. Their access to resources, knowledge, financial services, markets and more varies according to their socio-economic location in society and their ethnicity. At the same time, the findings suggest that a relatively simple empowerment process – training in wheat–maize technologies through a women’s organisation – has started to enable women to secure access to innovations and start making important choices in their lives. The fact that they have a strong woman as a leader appears pivotal to success. It is worth analysing and verifying this process through an empowerment conceptual lens. Kabeer (1999) argues that the ability to make choices is a defining feature of empowerment: to be disempowered implies to be denied choice. The concept of empowerment refers to the processes by which people who have been denied the ability to make choices acquire such an ability. Kabeer considers that the ability to exercise choice can be thought of in terms of three inter-related dimensions: Resources (preconditions) → Agency (process) → Achievements (outcomes). Resources include material, human and social resources which serve to enhance the ability to make choice. Agency is the ability to define one’s goals and act upon them. It can take the form of decisionmaking, bargaining and negotiation, deception and manipulation, subversion and resistance, and the processes of reflection and analysis. Taken together, resources and agency allow women to achieve outcomes important to them (Kabeer 1999).

Kabeer’s analytical framework (1999) is helpful to explore our findings further. She points out that the concept of agency has positive and negative meanings in relation to power. The negative sense of “power over” refers to the capacity of people (and organisations) to override the agency of others. To an extent, our data supports the enactment of this form of power through the RDRS (and its funders). Its targeting criteria, which are both “hard” (area of land farmed) and “soft” (the trainee should be known to us) exclude low-income women and men and thus disallow them the chance to express and build their agency. This may have the long-term effect of deepening their marginalisation. In the positive sense of “power to”, agency describes people’s capacity to define their own life choices and to pursue their own goals (Kabeer 1999). Our data supports this reading of agency as well. Some Santal and ethnic Bengali women are starting to define their own life choices and pursue their own goals – facilitated by the women’s organisation. A third form of agency, described as “power with”, refers to the capacity to augment power through collective action. There is evidence of this form of power being operationalised in the study community. Successful women innovators receive support from male family members and their extended family. Santal and Muslim women are working together through the Kalipara Union Federation to cooperate in securing access to wheat–maize innovations. Finally, power can also exist in the absence of any apparent agency. Norms and rules governing social behaviour can ensure that certain outcomes, such as identity-based marginalisation, are reproduced without obvious exercise of agency (Kabeer 1999).

The question arises as to the degree to which Santal women are able to use their personal agency to overcome the “power over” and the hidden forms of agency which combine to keep them left behind. An analytic model presented in the Introduction (see Figure A1) posited that four structural drivers underpin marginalisation processes: (1) an inadequate asset base; (2) poor access to services and infrastructure; (3) weak political voice, empowerment and institutional governance; and (4) identity-based exclusion and social norms. The first three structural drivers – assets, services and voice – can also act as “enablers” to lift people out of poverty. However, the processes of identity-based exclusion – and the social norms which underpin and “rationalise” this – are an underlying driver preventing marginalised people (including middle-income Muslim women) from accessing and using the first three drivers to their advantage.

Our data show that Santal women are beginning to flip the first three drivers of marginalisation to their advantage. They are using their agency to turn the tables on their identity-based exclusion through securing access to assets, services and institutional governance processes in relation to wheat–maize innovations, and that they have achieved this in a very short time. The data further shows that low-income Muslim women involved in field-based agriculture are seizing the opportunities first opened by the Kalipara Union Federation and Santal women in order to insert themselves into these empowerment processes. This includes setting up further women-led networks – for example, the low-income Muslim woman who organised a learning group of 25 women in her community. Whilst Muslim and Santal women do not share all the features of horizontal group-based forms of marginalisation (see Figures A1 and A2), they share sufficient features to enable cooperation along specific trajectories. For example, low-income Muslim and Santal women are engaged in field agriculture, have the same household responsibilities and the same interests in securing income, but both groups have long been excluded from training on field crops.

Taken together, this analysis suggests that the transformational power of women’s agency has been underestimated in conceptualisations of the drivers of horizontal group-based processes of marginalisation. This conclusion is summarised in Figure A3. It shows that the establishment of the Union Federation and the channelling of technical training to women was the precondition for women, Santal and low-income Muslim, to effectively express their agency. This in turn has led to the ability of Santal and low-income Muslim women to secure a range of achievements. In turn, this is starting to shift the balance of power towards women who have been left behind.

**Figure 2.**
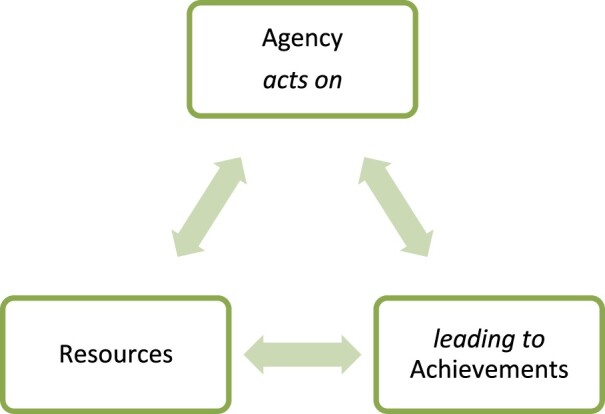
The power of women’s agency in creating positive feedback loops. Source: The authors.

It appears that even in circumstances of very low access to assets and services, weak political voice and identity-based marginalisation processes, women’s agency is a powerful mechanism for overturning all of these, to a greater or lesser degree. This allows us to modify Kabeer’s simple linear model with a more complex version (Figure 2). The two-way arrows symbolise what will become increasingly systemic iterations between agency, resources and achievements as feedback loops are set in motion. The challenge for agricultural research organisations and their partners is to feed into and reinforce these feedback loops in a positive way that speaks to the “power to” and “power with” forms of agency that we have described.

Finally, we should take note of the extraordinary intrinsic power poor people can have to change their lives. In so doing, they very much illustrate Sen’s (1990, 44) conceptualisation of empowerment as “replacing the domination of circumstances and chance by the domination of individuals over chance and circumstances”. This is one of the building blocks of achieving the ambition of the SDGs to leave no one behind.

## Supplementary Material

Online appendix

## Note

1 Due to limited data, the experience of Hindu women is not discussed in this article.
