# Supplementary material for: Leaving no one behind: how women seize control of wheat–maize technologies in Bangladesh
Source: Rev Can Etudes Dev. 2019 Aug 29;41(1):20–39. doi: 10.1080/02255189.2019.1650332 (PMC7077353; doi:10.1080/02255189.2019.1650332)
Supplement: Online appendix [file RCJD_A_1650332_SM0272.pdf]

Online Appendix

Leaving no one behind: how women seize control of wheat-maize technologies in Bangladesh

Cathy Rozel Farnworth, Tahseen Jafry, Siddiqur Rahman and Lone B. Badstue

Canadian Journal of Development Studies /Revue canadienne d'études du développement

Table A1. Themes for focus group discussions (FGDs) and semi-structured interviews (SSIs).

Tool	Respondent Category	Themes
Community Profile SSIs	Key informants	Overview of key features of the community and trends over the past 10 years: livelihood dynamics, marriage practices, educational opportunities, economic development and other.
Well-being FGDs	Low-income adults ages 25–55	Factors shaping socio-economic mobility, poverty trends and their gender dimensions.<br>This includes a "Ladder of Life" activity which has two objectives. First, wide-ranging criteria for determining wealth and poverty are agreed, and a scale of one to five (in the Bangladesh version of the exercise) created. Then households in the community are allocated to this scale thus creating rings on the "Ladder of Life". Second, the same exercise is repeated with respondents asked to discuss and present the situation 10 years ago. This provides the basis for a discussion on the causal factors of women and men moving in and out of poverty and how they relate to women and men's decision-making power and participation in innovations.
Gender Norms and Capacity to Innovate FGDs	Middle-income adults ages 25–55	Gender norms and household and agricultural / marketing roles; gender norms and household bargaining over livelihoods and assets; intimate partner violence; women's mobility.<br>Agency; community trends; enabling and constraining factors for innovation, and their gender dimensions; social cohesion, networks and social capital and their gender dimensions.<br>Some of this information is established through a "Ladder of Power and Freedom," which seeks to establish trends in self-perceived levels of decision-making currently and 10 years ago.
Innovator pathways SSIs	Recognised innovators	Explores in depth the trajectory of individual experiences with new agricultural practices, and the role of gender norms and capacities for innovation in these processes.
Life history SSIs	Key informants	To understand the life stories of men and women in the community who have moved out of poverty or remained trapped in poverty, and how gender norms, assets and capacities for innovation in agriculture shaped these dynamics.
Aspirations of Youth FGDs	Youth ages 16–24	To understand agency of young people in determining their life choices and their participation in innovation processes.

Source: Petesch et al. 2018.

Table A2. Training in wheat-maize innovations, by ethnicity and gender.

	Union Federation Membership (% of total)	Women trained via Union Federation	Men trained via Union Federation	Trained in wheat maize innovations via RDRS[a]	Percentage of all trainees trained
Santal Women	422 (55%)	45	-	-	12.0%
Santal Men	60 (7%)	-	5	-	1.3%
Bengali Muslim/ Hindu Women	344 (38%)	55		-	14.7%
Bengali Muslim/ Hindu Men	-	-	-	270	72.0%
Women (total)	766	100	-	-	26.7%
Men (total)	60	-	5	270	73.3%

Note: [a] RDRS= Rangpur Dinajpur Rural Service.

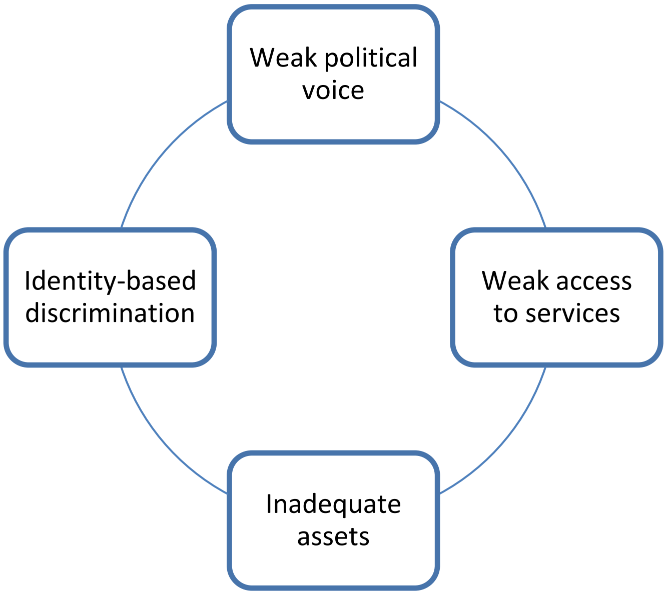

Figure A1. Structural drivers underpinning marginalisation processes.
Source: Adapted from Mittal, Pereram, and Korkeala (2016).

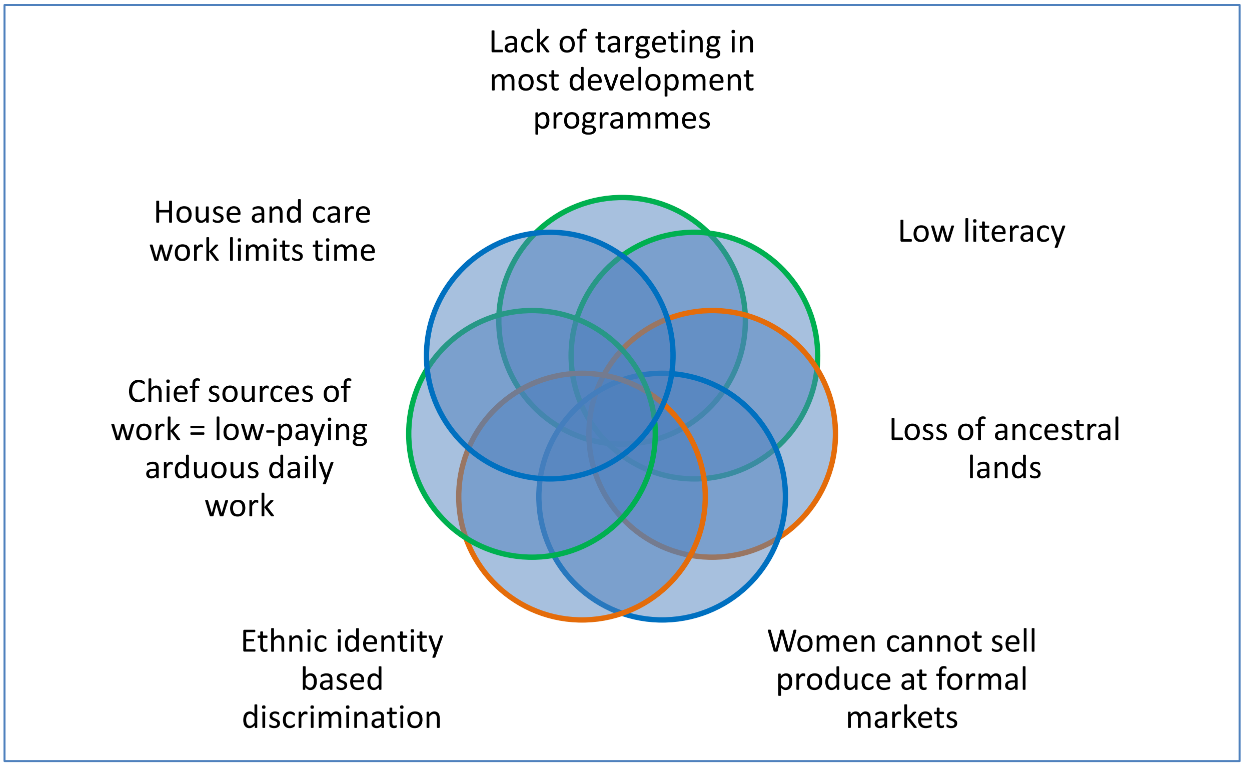

Figure A2. Overlapping drivers of Santal and ethnic Bengali women marginalisation.

Note to figure: Constraints that typically apply to ethnic Bengali women also apply to Santal women, such as time-consuming responsibility for household and care work, and an inability to sell agricultural produce at formal markets. These constraints are shown in blue. However, the position of Santal women is worsened through identity-based discrimination, the loss of land, general lack of targeting in development programmes and even lower literacy than the average for Muslim women. Constraints specific to Santal women are shown in orange. Low-income Muslim women share some constraints with Santal women, particularly engagement in low-paying day labour. The constraints shared by Santal and low-income Muslim women are shown in green. These drivers of marginalisation can act together to create a downward spiral, whereby negative outcomes cause further negative outcomes. The outcomes are unevenly distributed among women, with Santal women experiencing the most intense overlapping of drivers.

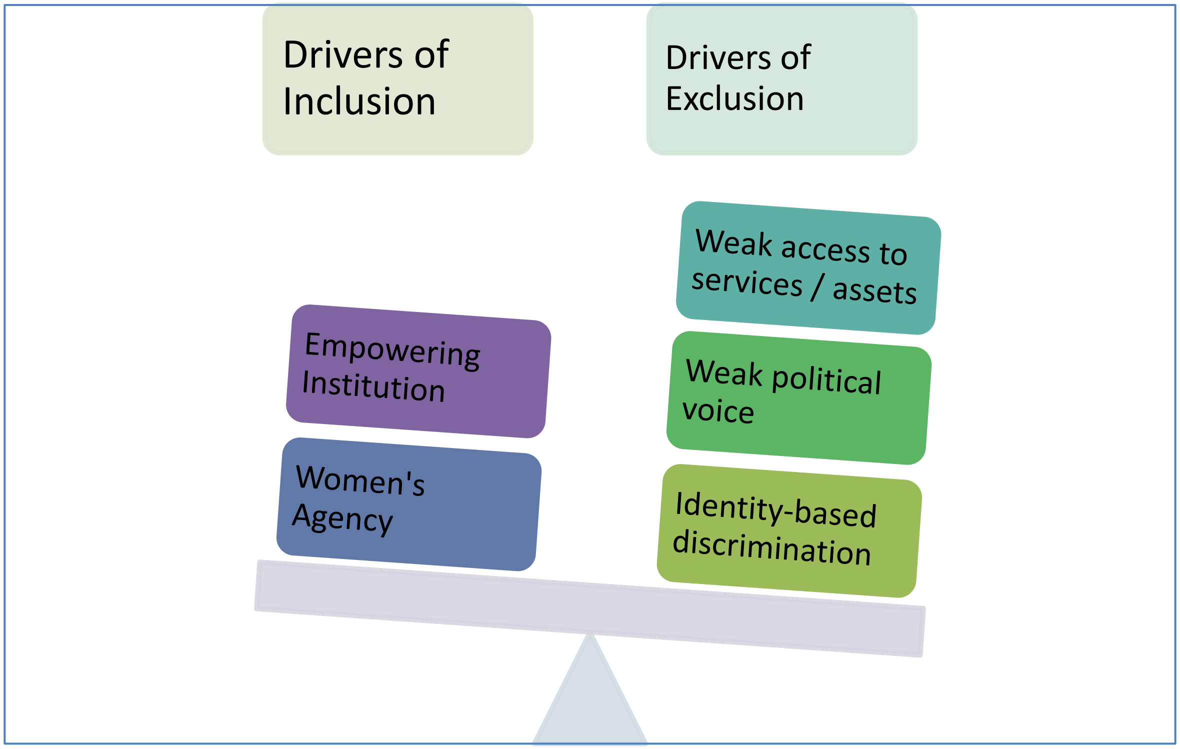

Figure A3. Rebalancing the drivers of exclusion.

References for the Online Appendix

Mittal, N., N. Pereram, and O. Korkeala. 2016. *Leaving No-One Behind in the Climate and Environment Context. Evidence on Demand*. London: DFID. https://www.gov.uk/dfid-research-outputs/learning-materials-leaving-no-one-behind-in-the-climate-and-environment-context

Petesch, P., L. Badstue, L. Camfield, S. Feldman, G. Prain, and P. Kantor. 2018. "Qualitative, Comparative and Collaborative Research at Large Scale: The GENNOVATE Field Methodology." *Journal of Gender, Agriculture and Food Security* 3 (1): 28–53.
